# Wettability, Adsorption and Adhesion in Polymer (PMMA)—Commercially Available Mouthrinse System

**DOI:** 10.3390/ma16175753

**Published:** 2023-08-22

**Authors:** Stanislaw Pogorzelski, Paulina Janowicz, Krzysztof Dorywalski, Katarzyna Boniewicz-Szmyt, Pawel Rochowski

**Affiliations:** 1 Institute of Experimental Physics, Faculty of Mathematics, Physics and Informatics, University of Gdańsk, Wita Stwosza 57, 80-308 Gdańsk, Poland; stanislaw.pogorzelski@ug.edu.pl (S.P.); p.janowicz.558@studms.ug.edu.pl (P.J.); krzysztof.dorywalski@ug.edu.pl (K.D.); 2 Department of Physics, Gdynia Maritime University, Morska 81-87, 81-225 Gdynia, Poland; k.boniewicz@wm.umg.edu.pl

**Keywords:** mouthrinse-PMMA, surface adsorption, surface thermodynamics, interfacial molecular partitioning, contact angle-wettability, adhesion, penetration-spreading kinetics

## Abstract

The study concerns the evaluation of the physicochemical and thermo-adsorptive surface properties of six commercially available mouthrinses, particularly surface tension, surface activity, partitioning coefficient, critical micellar concentration, Gibbs excesses at interfaces, surface entropy, and enthalpy. The aim was to quantify their effect on the adhesion and wettability of a model poly(methyl methacrylate) (PMMA) polymer. The adsorptive and thermal surface characteristics were derived from surface tension (*γ_LV_*) vs. concentration and temperature dependences. Polymer surface wettability was characterized by the contact angle hysteresis (*CAH*) formalism, using the measurable advancing *Θ_A_* and receding *Θ_R_* dynamic contact angles and *γ_LV_* as the input data. Further, wettability parameters: Young static angle (*Θ*), film pressure (*Π*), surface free energy (*γ_SV_*) with its dispersive and polar components, work of adhesion (*W_A_*), and adhesional tension (*γ_LV_* cos*Θ_A_*) were considered as interfacial interaction indicators. The mouthrinse effect demonstrated the parameter’s evolution in reference to the PMMA/pure water case: *Θ*, *Θ_A_* and *Θ_R_*↓, *CAH*↑, *Π*↓, *W_A_*↓, *γ_SV_*↓, and *γ_LV_* cos*Θ_A_*↑. Furthermore, the variations of the surface excess ratio pointed to the formation of multilayered structures of surfactants composing the mouthrinse mixtures considered. The contact angle data allowed for the penetration coefficient and the Marangoni temperature gradient-driven liquid flow speed to be estimated.

## 1. Introduction

Mouthrinses are frequently used in dentistry to improve dental health. They can be useful in preventing plaque accumulation by adding antibacterial compounds such as chlorhexidine. They are a very popular additional oral hygiene element, and there are plenty of individual products whose compositions are in a state of continuous modification. In order to be effective on smooth surfaces, a mouthrinse should spread completely over the tooth surface to enlarge its contact area and lower the contact angle (CA), which is attributed to its wettability [1]. Another important property is the capacity of a liquid to penetrate through pores and capillaries in hard materials of the oral cavity, as determined by its viscosity, surface tension, and contact angle on the capillary surface, expressed by the penetration coefficient (PC) [2] and Marangoni surface tension-gradient flow speed [3]. A typical mouthwash on the market is a water–glycerin mixture, consisting of an additional sweetener (saccharin), surfactant (PEG-40 hydrogenated castor oil, sodium lauryl sulphate, polysorbate 20, etc.), preservative (sodium benzoate), some dyes, and flavoring agent, as well as having two oral health substances, an anticaries compound (sodium fluoride) and an antimicrobial drug, as recently reviewed in detail [4]. The main aim of this concept research is to quantify the mouthrinse effect on adhesion and surface wettability of a model PMMA polymer/mouthrinse system. The interfacial interaction signatures in mouthrinse/oral solid surfaces are attributed to several physical mechanisms simultaneously taking place at the air/liquid and liquid/solid interfaces, namely surface adsorption, adhesion, and spreading, which are quantified in terms of several parameters determined from detailed adsorption and wettability studies performed on model systems. To address the problem, the first surface adsorptive and thermal parameters of six commercially available mouthrinse products were derived from the surface tension-temperature and surface tension-concentration dependences experimentally determined using the de Nuoy ring method [5]. To obtain the surface wettability parameters, the contact angle hysteresis (*CAH*) formalism was used, which is based on the three measurable quantities: dynamic contact angles: advancing (*θ_A_*), receding (*θ_R_*), and probe liquid surface tension (*γ_LV_*) [6]. Apart from the dynamic contact angles, the surface free energy of solids (*γ_SV_*), 2D adsorptive film pressure (*Π*), adhesional tension, works of adhesion (*W_A_*), and spreading (*W_S_*) were selected as condition level indicators of the studied PMMA-water (reference) and PMMA-mouthrinse-treated surfaces. Modern oral cavity dental materials include a wide group of polymers, ceramics, composites, metals, and their alloys. In this paper, a PMMA polymeric substratum of well-defined surface composition and properties, largely met inside the human mouth as a replica for teeth or material for preparing dentures, was used as the reference material. Polymethylmethacrylate (PMMA) is one of the most widely used acrylate plastics in light of its excellent biocompatibility and aging resistance. Since PMMA is a weakly polar polymer compound that contains –CH_3_, –CO, and –OCH_3_ groups, surfactant-containing liquids can interact with PMMA surfaces through a variety of adsorption mechanisms [7]. In the studied phenomenon, the essential problem seems to be bacterial attachment to surfaces. The biochemical studies highlight that the pellicle, which is a thin layer of organic material (saliva glycoproteins, phosphoproteins, proteins, enzymes, and receptors for bacterial adhesins), plays a crucial role. However, the physicochemical mechanisms of bacterial adhesion involve a thermodynamic model based on the interfacial free energies of liquids and interacting surfaces [8]. Moreover, the surface topography, together with surface roughness and surface pattern, influences cell adhesion. However, surface wettability’s energetic effect on solid surface bioadhesion had already been addressed. In particular, as demonstrated for polymers, values of water contact angles ranging from 40° to 70° are reported to be the most suitable for cell adhesion [8]. The surface free energy (SFE) decrease was observed for the coated surface with saliva in reference to the free one (from 36.6 to 31.8 mJ m^−2^) for PMMA acrylic resin [9]. Similarly, a decrease in SFE was also observed for denture acrylic resins after storage in substances for the hygiene of dentures [10]. Comprehensive CA studies of saliva and mouthwashes on enamel and composite dental materials were performed to understand how saliva interacts with restorative materials [11]. Water (over 99%) and inorganic and organic compounds such as immunoglobulins, proteins, enzymes, mucins, and nitrogenous compounds compose the majority of saliva. The interaction between saliva and morphological structures, mucosa, and hard dental tissues is critical for overall oral health and physiology. A balance of requirements often governs mouthrinse product formulation, including antibacterial, remineralization, and antidemineralization, stability, and taste requirements. In fact, these requirements are often conflicting, so it is not possible to point out the most effective mouthrinse product among the studied ones here. Consequently, further biological oral bacterial biotome destruction, physicochemical direct substrata surface dissolution, and erosion studies remain to be carried out that are correlated to the discussed here adsorptive, thermodynamic, and wettability signatures of a model mouthrinse/PMMA system useful to create a novel oral hygiene liquid composition selection procedure.

## 2. Theoretical Background

### 2.1. Adsorptive and Thermodynamic Surface Characteristics of Surfactant Solutions at Liquid-Vapor (LV) Interface

Surface tension vs. (bulk) concentration ($\gamma \left(c\right)$) plots for surfactant-containing systems can be analyzed in a wide concentration range, from the premicellar (c<cmc, where cmc stands for the critical micelle concentration) to saturation adsorption (c>cmc) regions, to obtain the interfacial adsorption parameters: Gibbs’ surface excess concentration *Γ*, molecular area *A_molec_*, surface activity σ=−dγ/dc, surface effectiveness $\gamma_{cmc}$ (the surface tension reduction at the so-called critical micelle concentration), and surface molecular partitioning coefficient of the surfactant molecules between the surface and bulk phase *K_P_*, as already performed in surface tensiometry studies on surfactant-preservative formulations for antimicrobial drug protection characterization [12]. Particularly, Gibbs’ equation quantitatively expresses the adsorption of surfactants at the liquid/vapor (*LV*) interface as follows [5]:

(1) $$\Gamma =-\frac{1}{RT}\frac{d\gamma }{dlnc}$$

where: *Γ* is related to the area per molecule by *A_molec_* = *N_A_/Γ*; *R*—the gas constant; *T*—the absolute temperature; and *N_A_* is the Avogadro number. The surface adsorption *Γ*, and further the molecular area *A_molec_* can be obtained from the slope of *γ* plotted versus the (natural logarithm of) concentration. Adsorption of a surface-active agent at the interface leads to the *γ* decrease as the total concentration is raised. At the particular point (and concentration = *cmc*), the interface becomes saturated with monomers ($\Gamma =\Gamma_{\infty }$). As a result, *γ* level off. The 2-D surface pressure of the adsorbed film is provided by $\pi =\gamma_{0}-\gamma$, where: *γ*_0_ and *γ* are the surface tensions of solvent and solution, respectively. The limiting area per molecule is given by $A_{lim}=\left(\gamma_{0}-\gamma_{cmc}\right)/RT$. Above a breaking point of $\gamma \left(c\right)$ dependence, the concentration range is named the micelle formation region.

The surface activity (*σ*) of a surfactant in the solution is proportional to the partitioning coefficient $K_{P}=\Gamma /c\;$of the surfactant molecules between the surface and bulk phases (c<cmc), as derived from Equation (1):

(2) $$\sigma =-\frac{d\gamma }{dc}=\Gamma \frac{RT}{c}$$

Assuming the conservation law for surfactant concentration, the total amount of surfactant molecules, *n* (moles), must follow the relation:

(3) n=AΓ+Vc

where *A* and *V* stand for the interfacial region area and the bulk volume, respectively [13]. It should be noted that intercomparisons of the surface rheological properties of surfactant films (in particular *Γ* and *A_molec_*) occurring originally at surfaces and these obtained from studies of an interfacial system covering a different area, performed on the same sample quantity of the bulk volume *V*, require the portioning effect of the surfactant molecules between the surface and bulk phases to be estimated since the geometric surface development coefficient *A*/*V* takes different values for the mentioned above systems. Such a correction is required for surfactant solutions with highly developed interfaces likely to be found in foam, emulsion, and other dispersive systems [14]. Surface tension versus temperature studies are important for the correction or calibration purposes of instruments and interfacial system data at different temperatures. The variation of *γ* for water with temperature *T* (in °C) is given by various investigators, but reliable data revealed the following dependence proposed by [15]:

(4) $$\gamma_{water}=75.67-0.14T-0.29\cdot 10^{-3}T^{2}$$

Thermodynamic surface parameters discussed here include the surface free energy *γ* (note that the surface free energy of liquids is commonly referred to as the surface tension), entropy (*S_S_*), and enthalpy (*H_S_*) of a model liquid interface. The surface entropy and enthalpy (per unit area) can be determined from the *γ* vs. *T* dependence [5]. The particular expressions are given by:

(5) $$S_{S}=-\frac{d\gamma }{dT}$$

(6) $$H_{S}=\gamma -T\left(\frac{d\gamma }{dT}\right)$$

Both relations are positive-valued due to the observable *γ* reduction with the temperature rise. As an example, in a pure water system, dγ/dT takes a negative value equal to −0.16 mN m^−1^ K^−1^; as such, *H_S_* > *Γ*. The entropic term in Equation (6) takes into account the energy losses related to the new surface creation. *H_S_*, being a temperature-independent parameter, is more informative of the two quantities, or at least it is unequivocally related to molecular arrangements at the interface affected by surface-active species adsorption, as found in seawater tensiometry studies [16].

### 2.2. Surface Wettability Energetics

The classic formulation of the force balance on the contact line, known as the Young–Dupre Equation, predicts the static, equilibrium (Young) contact angle *θ* in terms of the interfacial free energies of the three interfaces of the system [5]:

(7) $$\cos\theta =\frac{\gamma_{SV}-\gamma_{SL}}{\gamma_{LV}}$$

where subscripts *SV*, *SL*, and *LV* denote the interfacial free energies between solid/vapor, solid/liquid and liquid/vapor, respectively.

Most of the solid surface free energy determination formalisms are based on Young’s equation, employing equilibrium CA data [17]. In contrast, the contact angle hysteresis *CAH*-based model developed by Chibowski allows the solid surface free energy *γ_SV_*, and the related surface wettability energetic parameters of liquid–solid surface interaction to be derived from only three measurable quantities: the surface tension of probe liquid *γ_LV_*, and the dynamic contact angles: the advancing *θ_A_* and receding *θ_R_*, with $CAH=\theta_{A}-\theta_{R}$ [6]. This approach is one of the latest methods for calculating the surface free energies of polymeric materials. Unlike the other approaches, it takes into account adsorption at the solid-surface interface. The 2D tangential tension *Π* induced by the adsorbed film is defined as:

(8) $$\Pi =\gamma_{LV}\left(\cos\theta_{R}-\cos\theta_{A}\right)$$

As a result, the apparent surface free energy of a film-coated surface $\gamma_{SF}=\gamma_{SV}+\Pi$ reads as follows ($\gamma_{SF}\equiv \gamma_{SV}$):

(9) γSV=Π1+cosθA21+cosθR2−1+cosθA2 

The surface free energy dispersive component $\gamma_{SV}^{d}$ is defined as:

(10) γSVd=14γLG1+cosθA2

From the surface energetics point of view, forces of dispersive nature dominate in the polymer (PMMA)-water solution system, and the term $\gamma_{SV}^{d}$ accounts for up to 0.89–0.98% in the total *γ_SV_* [18].

The work of spreading *W_S_* of liquid on a solid surface can be easily derived from the work of adhesion *W_A_* ($=\gamma_{LG}\left(1+\cos\theta_{A}\right)$) and the work of cohesion *W_C_* ($=2\gamma_{LV}$):

(11) $$W_{S}=W_{A}-W_{C}$$

This thermodynamic quantity relates the wettability to the mechanical strength of adhesion and allows us to characterize the competition between solid–liquid adhesions with different liquids [19].

### 2.3. Surfactant Adsorption at Polymer Surfaces—Adhesional Tension

By referring to Equation (7), the difference between $\gamma_{SV}$ and $\gamma_{SL}$, given simply by $\gamma_{LV}\cos\theta$, is called the adhesional tension, which stands for another important quantity to predict the wetting properties of solid materials affected by surfactant adsorption. As found, there is a linear dependence between the adhesional tension and surface tension of aqueous surfactant solutions (c<cmc) [20]:

(12) $$\gamma_{LV}\cos\theta =a\gamma_{LV}+b$$

where *a* and *b* are constants. On the basis of such a linear relationship and relating Young’s equation with the Gibbs adsorption relation [21], the surfactant’s adsorption amounts at the interfaces can be obtained as follows [7]:

(13) dγLVcosθdγLV=ΓSV−ΓSLΓLV

where subscripts denote the corresponding interfaces. The relative adsorption at interfaces can be simply quantified upon analysis of the $\gamma_{LV}\cos\theta \left(\gamma_{LV}\right)$ plot. By assuming $\Gamma_{SV}=0$, the surface excesses ratio is provided by the slope a=ΓSLΓLV, as results from Equation (13).

## 3. Materials and Methods

Poly(methyl methacrylate) and PMMA (Organika S.A. Sarzyna, Nowa Sarzyna, Poland) square (20 mm × 20 mm) plates, selected as a model polymer substratum, were flushed in a 20% methanol solution in an ultrasonic cleaner for 15 min and rinsed with Milli-Q water. The procedure for preparation and cleaning of PMMA is presented in detail elsewhere [22]. This study evaluated the principal physical properties of six commercially available mouthrinses apart from Coca-Cola, orange, and apple juices (available from local markets) commonly found in the oral cavity, namely surface tension *γ_LV_*, density *ρ*, dynamic viscosity *μ*, acidity (pH), and liquid/solid contact angle (CA). The studied liquid’s surface tension was measured with a tensiometer (PI-MT1M, Donserv, Warsaw, Poland) based on the de Nuoy ring method (6 cm platinum–iridium ring, Sinterface, Berlin, Germany), with an accuracy of 0.2 mN m^−1^. The pH measurements of the model liquids were carried out with a pH-meter (CP-315, Elmetron, Zabrze, Poland) with a universal electrode. The Ubbelohde viscometer (Equimed, Krakow, Poland) was used for the kinematic viscosity measurements. Table 1 collects the physical and surface characteristics of the probe liquids. Listerine Cool Mint and Dentalux (+alcohol) were the most acidic liquids (pH = 4.3 and 4.9). Low pH values might stimulate the formation of CaF_2_, which is said to form a protective layer on enamel from which F^−^ ions are released [2]. In addition, low pH and high buffer capacity values seem undesirable, leading to enamel demineralization [1].

**Figure 1:**
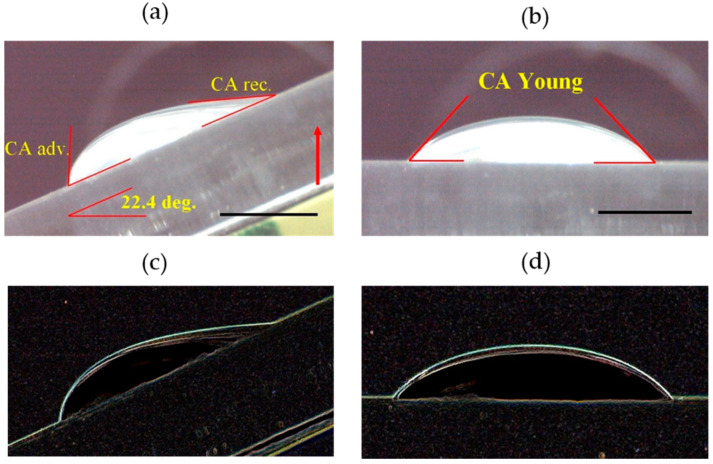
Exemplary images of a sessile drop shape as a basis for (**a**) dynamic and (**b**) Young equilibrium CA determination with the inclined plate method, for Listerine Cool Mint-PMMA system at 25 °C; *γ_LV_* = 37.6 mN m^−1^; bar = 2 mm. Images (**c**,**d**) are derived from (**a**,**b**), respectively, with FIND EDGES routine of ImageJ program, useful to sharpen the interfacial border line.

A circular (10 cm diameter) glass vessel containing the studied solution was temperature-controlled within the range 22–45 °C in order to obtain a surface tension-temperature dependence. Distilled water, used to prepare a diluted mouthrinse solution, was taken from a water deionization apparatus (Millipore, conductivity 0.05 μS cm^−1^) with pH 6.8 ± 0.1 and the surface tension *γ_LV_* = 72.5 ± 0.2 mN m^−1^ at room temperature *T* = 22 °C. CA measurements were performed on PMMA substrata enclosed in a humidity-controlled cell (see Figure 1 in [23]). To maintain saturation, the probe liquid was placed inside a temperature-controlled cell. A similar CA determination system based on the sessile drop geometry was already used in [24]. The axisymmetric sessile drop shape profile routine was used to evaluate CAs from the sessile drops (3–5 mm in diameter) of probe liquid images [18]. 4–7 CA measurements were performed at 5–10 different surface locations for the spatial inhomogeneity evaluations. The Young-equilibrium CAs were measured after 30 s from the moment of the liquid drop deposition from both sides of the image by means of the ImageJ program and then averaged (the obtained CA determination errors were ~1°). The CA hysteresis, *CAH*, was evaluated from the sessile drop shape taken with a tilted plate set-up for larger drop volumes (20–50 μL), as presented in [18]. Exemplary sessile drop images for Listerine Cool Mint/PMMA surfaces are depicted in Figure 1. Particular values: advancing CA $\theta_{A}$ = 65.2°, receding CA $\theta_{R}$ = 18.7°, *CAH* = 46.5°, plate inclination angle = 24.4°; Young CAs θ = 46.5° (left) and 45.6° (right), mean value = 46.1°. Small raindrops with a radius up to about 2 mm are nearly perfect spheres, but for a larger radius (exceeding the so-called capillary length *l_c_* = 2.7 mm for the air–water interface at 25 °C), they become increasingly flattened; the capillary length ($l_{c}=\sqrt{\gamma_{LV}/\rho g\;}$) is expressed via the liquid surface tension *γ_LV_*, density *ρ*, and gravity acceleration *g*.

There is a relation adapted here between the Young’s equilibrium contact angle and the dynamic contact angles: cos *θ* = ½ cos *θ_A_* + ½ cos *θ_R_*, experimentally verified for metallic surfaces covered with spread paint layers [25]. However, some more complicated relationships have also been postulated [26]. In order to obtain the surface tension *γ_LV_*-mouthrinse concentration dependence, surface tensions were determined on the subsequently diluted samples (of initial concentration—*c*_0_) and plotted as a function of the normalized (relative) concentration *c_n_ = c*_0_/*n*, where: *n* = 2, 3 …

## 4. Results and Discussion

### 4.1. Surface Adsorptive Parameters of Mouthrinse Solutions at Air/Water Interface

**Figure 2:**
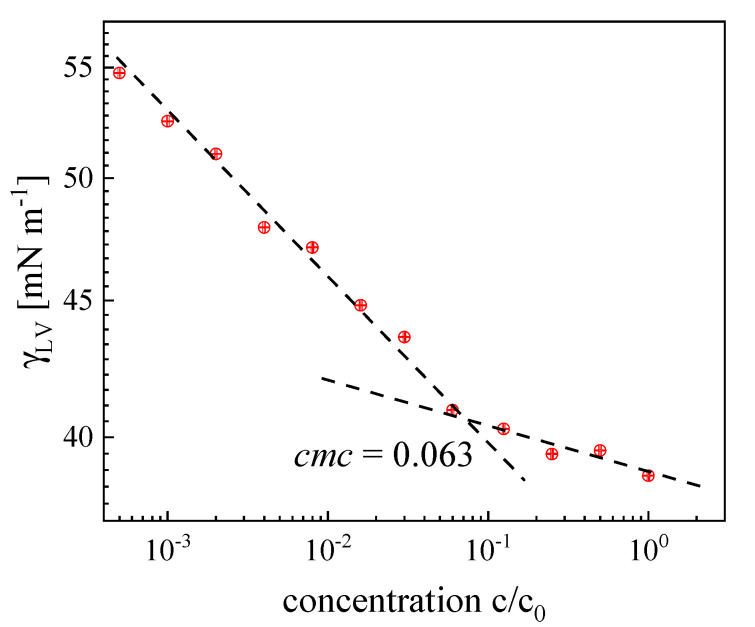
Surface tension of Xerostom solution vs. relative concentration *c/c*_0_ plot at *T* = 22 °C. The straight-line best-fit approximations to the data were used to determine the inflection point (*c/c*_0_ = 0.063, corresponding to *cmc*).

The principal parameters of the surface characteristics for mouthrinse formulations and other model substance water solutions, derived from a surface tension vs. concentration plot, as shown in Figure 2 for Xerostom, are collected in Table 2. In this system, a smooth discontinuity of the *γ*(*c*) plot can be first noticed (*c* < *cmc*; around 0.5) at the so-called critical aggregation concentration, followed by another one at *cmc*, where regular micellar structures are expected to appear. Such a scaling approach yields a universal concentration measure of a water mixture of largely unknown composition and surface activity in reference to the original (initial) sample concentration *c*_0_.

The lowest cmc value was noticed for Xerostom, whereas for the remaining mouthrinses it was contained within the range 0.03–0.12, still much lower than the original saliva-forming substance mixture (0.31). Mouthrinses cmc data revealed that the concentrations recommended by the producers *c*_0_ to apply in a patient’s practice turned out to be around 8–16 times higher than those required to obtain the saturated interfacial layer (at c=cmc). Under such a condition, the micellar structures are formed in liquid bulk and are capable of entrapping insoluble substances via the micellar solubilization mechanism [5]. As can be seen, the studied substances are capable of lowering the water surface tension (=72.5 mN m^−1^) by approximately 20–30 mN m^−1^.

It seems to be of interest to compare surface effectiveness *γ_cmc_* for a group of model surfactants used in physical chemistry studies. For example, anionic SDS (sodium dodecyl sulfate) lowers the water surface tension to ~44, cationic DTAB to ~35 and non-ionic C_12_E_m_ to ~32 mN m^−1^. Further adsorptive parameters: surface activity $\sigma =-d\gamma_{lv}/d\left(c/c_{0}\right)$ (obtained from the linear part of the $\gamma_{LV}\left(c\right)$ plot for c<cmc), and the corresponding *A_molec_* related to *Γ_max_* were determined as well. *Γ_max_* values remained in the range spanning from 0.24 to 0.94 ·10^6^ mol cm^−2^, which corresponded to the limiting *A_molec_* varying from 170.2 to 839.5 nm^2^ molec.^−1^, which is characteristic for so-called LE (liquid expanded) surface films [8]. *A_molec_* values registered for medical liquids (glycerol and glycols) turned out to be lower by a factor of 10^2^ (0.58–1.57 nm^2^ molec.^−1^ [27]). Saliva-formed films revealed *A_lim_* (=12.6 nm^2^ molec.^−1^), i.e., 10 times lower than the mouthrinse did on average. From a medical point of view, it is particularly important to attain a concentration of free preservative agents (included in mouthrinse formulations) in water high enough to achieve sufficient antimicrobial protection. It seems clear that preservatives may remain more or less available for contact with microorganisms depending on their location, i.e., air-water, solid water, or bulk water phase. The surface activity of the surfactant *σ* is proportional to the partitioning coefficient $K_{P}=\Gamma /c$ of the surface-active species between the air/water and bulk phases. Among the studied mouthrinses, the most surface-active agents were Eurodont and Dentalux (the lowest cmc and *γ_cmc_*, and the highest |*σ*|). In addition, the highest absolute values of *σ* indicate that the mentioned substances molecules are most hydrophobic and slightly soluble in bulk. Our previous surface tensiometry studies on formulations of surfactants with preservatives revealed, in particular, that methylparaben has |*σ*| value about 10 times lower than benzalkonium chloride (BAC) and is more soluble in the aqueous phase in the form of free molecules, making it more available for contact with microorganisms residing therein [12].

### 4.2. Surface Thermodynamics Characteristics

**Figure 3:**
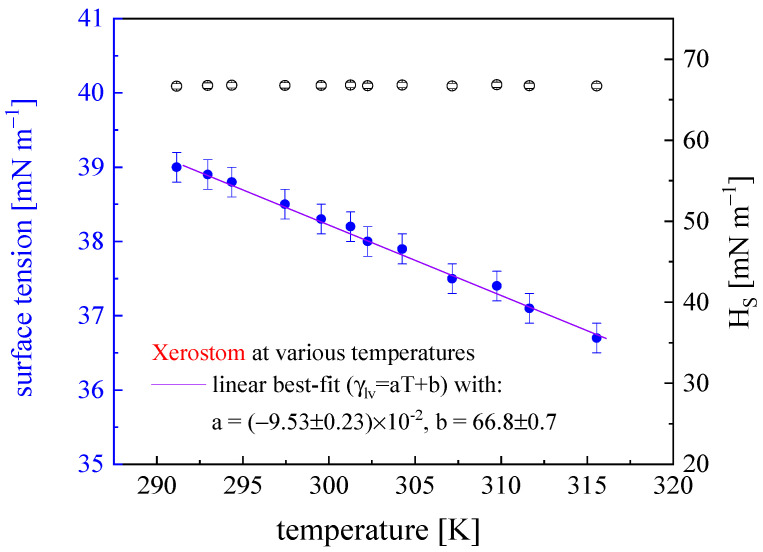
Surface free energy *γ_LV_* and enthalpy *H_S_*, for Xerostom solution (at *c = c*_0_). *S_S_* (= 0.09 ± 0.02 mN m^−1^ K^−1^) obtained from the slope of the *γ_LV_*(T) relation linear fit.

An exemplary surface tension *γ_LV_* versus temperature T dependence, together with *H_S_* thermodynamic potential variability, obtained for Xerostom, is shown in Figure 3. A complete set of surface thermodynamic functions for the studied liquids is collected in Table 3. The observable quasi-linear *γ_LV_*(*T*) dependence can be approximated with a function *γ_LV_* = −0.095*T* + 66.8. *H_S_* remains almost constant, slightly leveling down as T increases. *S_S_* (values from the *TSs* column (Table 3) divided by *T* = 295 K) took values ranging from 0.10 to 0.23 mN m^−1^ K^−1^, comparable to the ones found for juices, pure water (0.13–0.18), and saliva (0.11). Higher values of *S_S_* point to a less complex surface layer structure consisting of adsorbed surface-active agents and counter ions present in the sub-layer water phase. A double electric layer likely to be present here forms an interfacial system of particular thermodynamics (of higher work of cohesion, *W_C_* = 2 *γ_LV_*) [28].

Surface tensions of the mouthrinses (= 31.7–39.9 mN m^−1^) are significantly lower than values characteristic for pure water (72.5), its water solutions (61.1–64.1), and saliva (53.6). It is known that the surface active substance adsorption leads to the surface tension drop $d\gamma_{LV}=\Gamma d\mu$, where *μ* is the chemical potential related to the concentration via dμ=RT lnc, while the Gibbs surface excess $\Gamma \propto d\gamma_{LV}/RT$ (see Equations (1) and (2)) for a gaseous surface film according to the Langmuir approach [5]. Surface entropy decreases with an increase in surface adsorption of the amphiphiles [29]. In other words, the surface adsorption from the bulk solution causes a larger negative entropy change.

Generally, *H_S_* took lower values for the mouthrinses (66.8–97.6 mN m^−1^) in reference to pure water (118.8), their derivatives (99.1–116.8 mN m^−1^), and saliva (85.6) as well. It can be noted that the entropic term *TS_S_* (related to the formation of the interfacial surface) contributions to the surface enthalpy *H_S_* varied from 0.42 to 0.68, being higher if referred to the pure water reference (0.39), reflecting the presence of adsorbed species. Lower *TS_S_* contributions were also noticed for other water-based liquids (0.38–0.41). In the studied water solution mixtures, besides proteins, carbohydrates, and glycerides, which are characterized by small surface activity, there are also very active free fatty acids, active esters, and alcohols. Their relatively small presence can strongly affect the resultant surface tension of a multicomponent film. In such highly structured, polymer-like interfacial systems, the thermodynamic and kinetic processes appear to be more complex [30]. It is interesting to note that the adsorption process is entropy-controlled (*TS_S_* > *γ_LV_*) for Listerine, Dentalux (+alcohol), and Dentalux (alcohol free), but the opposite situation, i.e., (*TS_S_* < *γ_LV_*) was evidenced for Xerostom, pure water, its derivatives (Coca Cola, orange juice), and saliva. Eventually, Ortho Salvia Dental and Eurodont formed adsorptive layers with *TS_S_*~*γ_LV_*.

### 4.3. Wettability Energetics from the Contact Angles Analysis

By referring to Table 4, the value of the static equilibrium contact angle *θ* for the reference system PMMA–pure water (=64.7°) is significantly higher than for the other probe liquids by about 20°, varying in the range of 30.2 (Eurodont) to 46.1° (Listerine); as such, the systems can be considered hydrophilic (*Θ* < 90°). A decrease in *θ* and *γ_SV_* observed as the PMMA is successively exposed to mouthrinses results from a few effects: adhesion of the liquid compounds, irreversible adsorption in solid pores, chemical erosion of polymeric surfaces, etching and micro-roughness creation, leaching of the more soluble PMMA components, etc. The remaining probe liquids (juices, Cola, and saliva) possess higher both *θ* and *γ_SV_* comparable to the pure water reference case. Generally, values of *γ_SV_* point to the low energy surface of the PMMA, ranging from 38.9 to 22.4 mJ m^−2^ in contact with pure water and Dentalux (alcohol-free), respectively. Such surface energies are characteristic of hydrophobic surfaces like polymers (20–30 mJ m^−2^). For all the studied water solutions, which mimic liquids found in the oral cavity, an increase in exposure time leads to the same trend: *θ*↓, *γ_SV_*↑, and *W_A_*↑ i.e., evidence of transition processes taking place at the solid/liquid interface (research in progress).

The contact angle hysteresis approach provides several further parameters, collected in Table 4, for the PMMA-probe liquid system that are useful in quantitative evaluations of surfactant-containing liquid/hydrophobic solid surface interactions. As a matter of fact, contact angle hysteresis parameter variability (referred to the clean, unaffected surface case) rather than their absolute (particular) values stand for a useful tool for the PMMA surface adsorption modification tracing after exposure to mouthrinse solutions. For the pure water at the PMMA surface, *CAH* = 24 ± 2.0° was similar to the one reported in the literature (=23.5 ± 1.5° [31]). There are several reasons responsible for the hysteresis effect occurring jointly: surface microroughness, chemical heterogeneity, liquid drop size effect, molecular reorientation of the adsorbed surfactant molecules, liquid penetration into solid pores, sandwich-like structures of the interfacial layer, etc.

**Figure 4:**
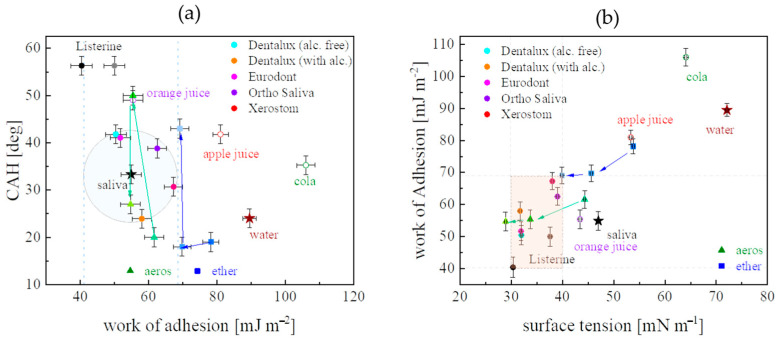
(**a**) Contact angle hysteresis (*CAH*) as a function of work of adhesion (*W_A_*); (**b**) *W_A_* plotted versus probe liquid surface tension (*γ_LV_*), for PMMA-probe liquid systems at 22 °C (295 K). Additional points (data from [32]): pure surfactant water solutions of nonionic Polyoxylene Lauryl Ether (blue) of a variable concentration increasing from 3.9 × 10^−4^ → 2.0 × 10^−3^ → 2.0 × 10^−2^ mol., and anionic Aerosol OT (green) with the concentration varying from 1 × 10^−2^ → 6 × 10^−2^ → 2 × 10^−1^ mol.

*CAH* values for the mouthrinse-treated surfaces are rather high, ranging from 23.9 (Dentalux + alcohol) to 46.6° (Listerine), which exhibits a surface activity diversity of the surface-active species forming the mixture since *θ_A_* (42.2–65.2°) corresponds to the most hydrophobic component, whereas *θ_R_* (12.1–22.3°) points to the less hydrophobic one. 2-D film pressure *Π* (4.9–19.8 mN m^−1^) was several times lower than recorded for the PMMA surface contacted with saliva, pure water, and water-originated liquids (20.3–29.2 mN m^−1^) which seems to be an effect of competitive surface adsorption at the PMMA/liquid interface. A water solution of mouthrinse spreads the PMMA surface to a greater extent (lower *Π* and *W_A_*) and is capable of covering, on the basis of competitive adsorption rules, the already covered surface with less surface active species. It makes the surface adsorption of mouthrinse solutions on the PMMA substratum a process favored energetically. Such a product can effectively remove already-adsorbent surface contaminants occurring on the dental material. As: $W_{A}=\gamma_{LV}+\gamma_{LV}cos\theta_{A}$, the second term, i.e., the adhesional tension contribution to *W_A_* (given by the ratio:$\;\gamma_{LV}cos\theta_{A}/W_{A}$), spanned from 0.29 (Listerine) to 0.45 (Dentalux + alcohol), and attained only 0.11 and 0.02 for pure water and saliva, respectively. Such a surface-energetics-derived parameter seems to be a signature of the adhesive strength removal ability of the mouthrinse liquids. The differentiated polarity of the probe liquids in contact with the PMMA surface is reflected in the distribution of the experimental points placed in the 2D space of *CAH* plotted versus *W_A_* and of *W_A_* versus *γ_LV_*, as depicted in Figure 4a and 4b, respectively.

**Figure 5:**
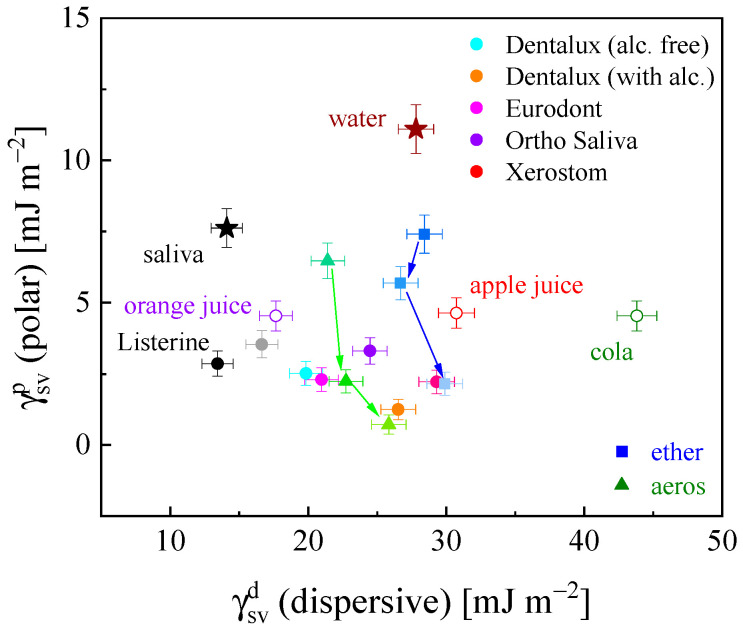
Polar $\gamma_{sv}^{p}$ versus dispersive $\gamma_{sv}^{d}$ terms of the total surface free energy $\gamma_{sv}$, for the studied water solutions in contact with PMMA substratum at *T* = 22 °C. Concentration evolutions for ether (green) and aerosol (blue) as in Figure 4.

As depicted in Figure 4a, a circled, shaded region covered an area *CAH* of 23.0–42.5° and *W_A_* of 42.1–67.8 mJ m^−2^, corresponding to the data for the studied saliva and mouthrinse-mediated PMMA surfaces. Other water-derived mixtures (water, juices, and Coca-Cola) and pure one-component surfactant solutions (despite their polarity and concentration) demonstrated significant point scatter outside the specified region that pointed to the particular adhesive signatures of the mouthrinse formulations. Similarly, in Figure 4b, the mouthrinse-corresponding points were contained in a narrow shaded box limited by *W_A_* ranging from 41.2 to 69.2 mJ m^−2^ and *γ_LV_* varying between 29.8 and 40.1 mN m^−1^, presented on the remaining background data located far outside this specific region. Finally, the particular values of *CAH*, *W_A_* and *γ_LV_* established in contact angle studies can be of use in the selection of the most proper and specific model liquids for further dental material wettability adjustment recommendations. From the surface energetics point of view, in the PMMA/water solution interactions, forces of a dispersive nature prevail, where the dissipative term $\gamma_{SV}^{d}$ in the total solid surface free energy *γ_SV_* accounts for up to 89–98%. The polar and dispersive terms of the total surface free energy for the PMMA surface affected by the studied liquids are depicted in Figure 5. Rather low $\gamma_{SV}^{p}$ values were obtained for mouthrinses (1.29–3.56 mJ m^−2^), significantly lower than those exhibited by the remaining liquids (4.54–11.1 mJ m^−2^). The dispersive term $\gamma_{SV}^{d}$ values were contained within a narrow range of 18.92–29.31 mJ m^−2^. The alcohol addition to the Dentalux mixture resulted in a lowering of $\gamma_{SV}^{p}$ and in an increase of $\gamma_{SV}^{d}$. The concentration increase of the standard surfactant solutions, both non-ionic (ether) and anionic (aeros), evidenced a significant drop of the polar component, from 7.3 and 6.2 mJ m^−2^ to 2.4 and 1.3 mJ m^−2^, respectively. At the same time, the dispersive term increased slightly from 26.8 and 22.3 to 30.0 and 26 mJ m^−2^, respectively. In general, the behavior of surfactants at the interface is determined by a number of forces, including electrostatic attraction, covalent bonding, hydrogen bonding, and the solvation of various species. Steric forces arise when molecules with long chain segments are present in the system, e.g., surfactants or polymers.

Generally, it was demonstrated that the *CAH* is neither correlating to wettability nor adhesion by showing the lack of correlation between the difference *θ_A_*–*θ_R_* and *θ*, and the pull-off force (adhesion). Overall results indicate that the *CAH* originates from a single source: the difference in liquid−solid interaction in the vicinity of the contact line during receding and advancing movement modes [33]. Hysteresis of the Cassie−Baxter wettability state (likely to be present here) depends on *θ_R_*; the smaller *θ_R_*, the larger *CAH*. Moreover, *θ_A_* is independent of the solid area fraction, and *θ_R_* decreases and the hysteresis increases as the solid area fraction increases. The large hysteresis is indicative of stickiness. The increased interactions at the water–adsorbed film interface drag the receding contact line, resulting in smaller *θ_R_* and larger *CAH*. It is important to point out, that the *CAH* has two components (*θ_A_* and *θ_R_*). Thus, if the *CAH* is dominated by the liquid−solid interaction at the interface, it will manifest as adhesion. In fact, adhesion correlates to *θ_R_* alone [34]. Moreover, only a surface modification process accompanied by a decrease in system energy can occur thermodynamically. In surfactant solutions, the changes in CA depend on the orientation of surfactant molecules on a surface. For a strongly hydrophobic surface, it is usually accepted that the hydrocarbon chain of a surfactant faces the surface, leaving the polar group exposed to water and thus causing a reduction in contact angle. Therefore, the interaction between a hydrophilic part of a molecule and the PMMA occurs not through direct contact between the two entities but via a layer of water film sandwiched in between. Since water could not adhere to, or spread over, the PMMA, i.e., water is mobile or slips on the hydrophobic-group exposed surfaces. As a result, the surfactant molecules are oriented on the PMMA surfaces in such a way that the hydrocarbon chains face the surface with the polar groups facing water, thus reducing the contact angles with increasing surfactant concentration (see Figure 12 in [35]). It should be noted that on the PMMA surface, the –CO, –OCH_3_ and –CH_3_ groups are present [36]. Therefore, surfactants can interact with PMMA surfaces through a variety of adsorption mechanisms. Oxygen in the –CO and –OCH_3_ groups can behave as an electron-donor in contact with water or other liquids having active hydrogen, but hydrogen in –CH_3_ has a small tendency to behave as an electron-acceptor and in some cases only very weak hydrogen bonds can be formed [36]. For this reason, PMMA is called a monopolar solid, in which the contribution of the electron-donor interaction in the adhesion of the liquid to the PMMA surface plays an important role. It is reflected in the surface free energy of PMMA, its components, and related adhesion parameters.

### 4.4. Wettability Characteristics vs. Bacterial Adhesion

For effective polymer surface preparation in order to avoid bacterial adhesion, multiple aspects of material properties should be considered, such as surface free energy, wettability, surface charges, and material surface architecture, as reviewed in [37]. In particular, surfaces with moderate wettability are more capable of binding bacteria or cells if referred to as extremely hydrophobic or hydrophilic surfaces. Water contact angles of 40–70° turned out to enhance cell adhesion and growth, whereas surfaces with CA of 54–130° had a higher adsorption of bacterial peptidoglycan. A hydrophobic substratum material with a surface energy of 20–30 mJ m^−2^ produced lower bacterial adhesion as compared to materials with higher surface energies. It was found that the polar component of the surface energy is important for cell adhesion and spreading, where a polar component lower than 5 mJ m^−2^ led to reduced cell spreading and a polar component greater than 15 mJ m^−2^ promoted spreading. It should be noted that a superhydrophobic (CA > 150°) surface can be prepared by enhancing material roughness to reduce the apparent surface energy, and because of this roughness, air becomes entrapped between roughening features when a liquid is in contact with the solid surface. Thus, the maintenance of entrapped air plays a crucial role in suppressing bacteria adhesion by reducing the contact area at the interface and further decreasing the adhesion force. The area fraction (f) where a probe liquid is in contact with a solid surface as opposed to air can be derived from the apparent contact angle ($\theta_{CB}$), assuming the Cassie-Baxter wetting state, and the Young contact angle θ at a smooth surface: $\cos\theta_{CB}=f\left(\cos\theta +1\right)-1$, as shown in exemplary water contact angle studies of polystyrene samples with varied surface wettability resulting from the plasma substrate treatment [37]. As a result, the surface energy was substantially increased, especially in the polar component, and the dispersive component was decreased significantly.

### 4.5. Mouthrinse Dissolution Effect on PMMA Wettability

The original mouthrinse product (Eurodont) of initial concentration ($c_{0}$) was subsequently dissolved with ultrapure water and then used in CA PMMA surface wettability studies. Wettability parameters for the system are collected in Table 5. First, the normalized concentration of saturation adsorption cmc (=0.032 $c_{0}$) was determined from the *γ_LV_*($c/c_{0}$) plot, as already given in Table 2.

**Figure 6:**
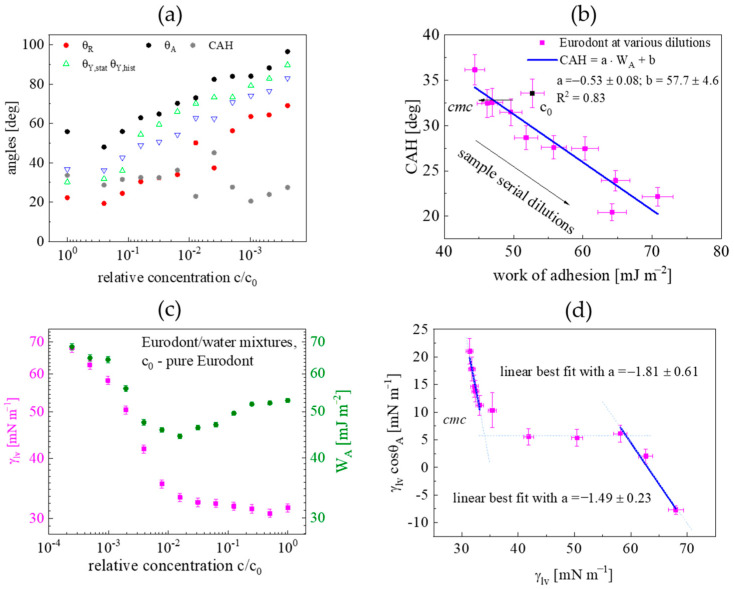
Wettability studies of PMMA-Eurodont system at T = 22 °C. (**a**) Static, dynamic CA and *CAH* versus relative concentration $c/c_{0}$; (**b**) *CAH* as a function of *W_A_* for serial Eurodont dilutions; (**c**) *γ_LV_* and *W_A_* plotted versus relative concentration $c/c_{0}$; (**d**) adhesional tension vs. surface tension (the $\gamma_{LV}cos\theta_{A}\left(\gamma_{LV}\right)$ dependence), leading to the surfactant excesses ratio: $\Gamma_{SL}/\Gamma_{LV}=a$ (slope) determination.

Values of *θ*, *θ_A_*, *θ_R_*, *CAH*, and *Π* as a function of the normalized concentration $c/c_{0}$ are shown in Figure 6a. As expected, for a regular surface-active compound mixture, in the submicelle region (c>cmc) no significant CA variability was observed. For concentrations lower than cmc, a continuous increase of static and dynamic CA was observed. However, in this region, *CAH* fluctuated around ~20.5–27.6°; similarly, the 2D adsorptive film pressure (*Π*) took significantly, i.e., two times higher (~27.7–32.0 mN m^−1^) values in reference to the sub-micellar region. This phenomenon results from the complex response of the already adsorbed molecular film structure to the adhesion tension ($\gamma_{LV}cos\theta_{A}$) induced by the mouthrinse application. *CAH* versus *W_A_* dependence, plotted in Figure 6b, can be approximated by a linear function (*R^2^* = 0.83) as *CAH*↓ and *W_A_*↑ with serial dilution and liquid polarity. The concentration dependence on both *γ_LV_* and *W_A_* is depicted in Figure 6c. A continuous decrease of both quantities was observed in the sub-micellar range (c<cmc), but *γ_LV_* attained almost a constant value (*γ_cmc_*~32.4 mN m^−1^) whereas *W_A_* further increased in the post-micellar concentration range (c>cmc). Since *W_A_* is given by the sum of the surface tension and the adhesional tension, the observed *W_A_* (under the condition *γ_LV_*~const for c>cmc) could result from the different multilayered structure organization of the adsorbed film (see Figure 7 in [7]). The slope of the $\gamma_{LV}\cos\theta_{A}\left(\gamma_{LV}\right)$ dependence, depicted in Figure 6d, allowed the surface surfactant excess ratio: $\Gamma_{SL}/\Gamma_{LV}$ to be determined (see Equation (13)). The excesses ratio derived for pre-micellar and post-micellar concentration ranges by means of a linear best-fit procedure was equal to –1.81 ± 0.61 and −1.49 ± 0.23, respectively. The conventional surfactants exhibit negative slopes (from −0.33 to −0.17), which indicates that they adsorb at the PMMA-water surface via hydrophobic interactions [38].

### 4.6. Mouthrinse Surfactants Partitioning between LV and SL Interfaces

The $\Gamma_{SL}/\Gamma_{LV}$ ratio, i.e., the surface surfactant excess ratio, was derived for all the studied mouthrinse solutions over the whole concentration range from the slope of the linear fit to the $\gamma_{LV}\cos\theta_{A}\left(\gamma_{LV}\right)$ dependence plots. The obtained values for the selected mouthrinse formulations, together with the data reported by others for the surfactant water solutions of differentiated polarity in contact with PMMA substratum, are collected in Table 6.

In general, the $\Gamma_{SL}/\Gamma_{LV}$ ratio for conventional surfactants such as non-ionic surfactant TX-100, Tween 20, anionic surfactant SDS, and cationic surfactant CTAB are all negative, demonstrating that they adsorb at the PMMA-water interface via hydrophobic interactions. Meanwhile, the conventional surfactants exhibit an absolute slope value of about 0.3, which indicates that they are likely to tile at the PMMA–liquid interface with about 1/3 of the adsorption amounts at the air–liquid interface. In particular, the slope of the linear dependence between the adhesional tension and surface tension is higher than −1 (from −0.33 to −0.17, see Figure 2 in [38]). As it was found, the effect depends on the kind of binary surfactant mixture and its composition. The surface excesses ratios measured here were all negative and contained a range from −1.49 (Eurodont) to −0.20 (Ortho Salvia Dental) at pre-micellar concentrations. Generally, the slope ($=\Gamma_{SL}/\Gamma_{LV}$) values were lower than −1, which is evidence of a higher surface concentration of the adhered material at the *S*/*L* interface than at the *L*/*V* one. Since the mouthrinse is a mixture of several compounds of differentiated surface activity, vertically complex sandwich-like multi-layered molecular structures could be formed, as suggested in [7]. Moreover, zwitterionic surfactants with hydrophilic polar heads containing both a positive and a negative charge show unique adsorptive properties on a solid substratum. For instance, betaines are an important kind of zwitterionic surfactant with high surface activity that could be found in several mouthrinse formulations [20]. Since in the literature it is suggested that the –CO, –OCH_3_ and –CH_3_ groups are present at the PMMA surface, for betaines, more than one adsorption interaction between surfactant molecules and the PMMA surface will appear: at low bulk concentration, the betaine molecules adsorb at the PMMA-liquid interface mainly through hydrophilic interaction with alkyl chains oriented towards the solution, which results in a decrease in adhesional tension. However, at concentrations far higher than cmc, more betaine molecules adsorb onto the solid surface by hydrophobic interaction and make the solid more hydrophilic, which would improve adhesional tension. As a result, the adsorption behavior between the conventional surfactants and the extended surfactants is quite different [9].

### 4.7. Penetration Coefficient and Marangoni Spreading Speed

The penetration coefficient PC is a measure of the ability of a liquid to penetrate into a capillary space, such as interproximal regions, gingival pockets, and pores. PC can be evaluated from the surface tension *γ_LV_*, contact angle *θ*, and viscosity *μ* of the mouthrinse-PMMA surface system as follows [2]:

(14) $$PC=\frac{\gamma_{LV}}{2\mu }cos\theta$$

A liquid with a low viscosity, high surface tension, and low contact angle (i.e., good wetting) could penetrate rapidly into the capillary-structured space. As reported from the in vivo CA studies of the mouthrinses on tooth surfaces (ranging from 37.0 to 54.0°), PC values could be extremely high for water (=0.241 m s^−1^) [1]. PC values determined in this research for the model mouthrinses are collected in Table 7. They range widely from 9.8 (Xerostom) to 6.5 (Listerine) 10^−2^ m s^−1^. It can be noted that the surface tensions of the rinses lie in the range between 39.9–29.7 mN m^−1^, *θ* vary from 21.9 (Dentalux-alcohol-free) to 46.9° (Listerine), and their viscosities are all higher than water. It was confirmed that the PC value for pure water is very high, much higher than demonstrated by water solutions (Coca-Cola, juices) and natural saliva (of higher *γ_LV_*) found in an oral cavity. Xerostom and Ortho Salvia Dental exhibited the highest PCs, whereas Listerine revealed the lowest PC value. This result shows that it would be worthwhile to modify mouthrinse formulations to increase their penetration efficiency. It should be noted that the dynamic contact angle *θ_A_* measured at the forward face of the moving liquid front can differ from the equilibrium CA, *θ*, depending on the capillary number ($Ca=u\mu /\gamma_{SL}$, where *u* is the specific rate of movement of the liquid-solid contact line front [42]), and expressed by the following empirical relation [43]:

(15) $$cos\theta_{A}=cos\theta -2\left(1+cos\theta \right)Ca^{0.5}\;$$

A spontaneous flow toward regions of high surface tension, so-called Marangoni flow, is another transport process taking place in a liquid layer placed at the solid surface, whose speed *U_S_* is attributed to the surface tension gradients [44]. The shear stresses appearing at the interface resulting from horizontal surface tension gradients are assumed to be equalized by viscous stresses under steady-state hydrodynamic conditions [45]. For the two-dimensional coordinate model, where x and z are horizontal (along the flat solid surface) and vertical coordinates, respectively, one obtains [46]:

(16) $$\mu \frac{\partial U_{S}}{\partial z}=-\frac{\partial \gamma }{\partial T}\frac{\partial T}{\partial x}-\frac{\partial \gamma }{\partial c}\frac{\partial c}{\partial x}=S_{S}\frac{\partial T}{\partial x}+\sigma \frac{\partial c}{\partial x}.$$

The outermost surface linear velocity resulting only from the temperature gradient parallel to the spreading flow direction for the liquid layer of thickness d can be expressed by the simplified theoretical relation [47]:

(17) $$U_{S}=\frac{d}{4\mu }S_{S}\frac{\partial T}{\partial x}.$$

In order to evaluate the thermocapillary flow velocity, the following assumptions were made: *d*—the sessile drop height was taken from the drop images (with the arbitrary fixed drop footprint radius r = 3.5 mm, directly related to *θ*). Values of d ranged from 1.87 (Dentalux + alcohol) to 2.67 mm (Listerine), being higher for saliva, water, and the remaining water solutions. The temperature difference between the rinse (*T* = 20 °C) and the oral cavity (=37 °C) likely to be present along the solid material at a distance Δx = 10 mm leads to the temperature gradient *∂T*/*∂x*~1.7 K mm^−1^. The supplementary physical and thermal surface parameters, i.e., *μ* and *S_S_*_,_ originate from Table 1 and Table 3, respectively. *U_S_* values are collected in Table 7 for the model liquids. Generally, Marangoni’s spreading mechanism is slower than the penetration one, with *U_S_* varying from 0.46 (Dentalux-alcohol-free) down to 0.20 cm s^−1^ (Listerine), i.e., being 16–47 times lower than PC values. Systematically higher *U_S_* were found for pure water, juices, Coca-Cola, and saliva (in the range from 0.39 to 0.80 cm s^−1^). It should be noted that apart from the thermal Marangoni effect, there is also the classic Marangoni one, driven by the surfactant concentration gradients over the solid substrata in the oral cavity (the second term of the right-hand side of Equation (16)). However, the surface activity σ (see Equation (2)) of a surfactant solution was determined in the framework of this study, and the surfactant concentration gradient (=∂c/∂x) is an undetermined quantity hardly to be estimated. In addition, it was demonstrated in thermo-elastic studies of natural surfactant films in Baltic Sea coastal waters that the principal role is played by the temperature-over-surfactant-mediated gradient effect [47].

## 5. Conclusions

Adsorptive and thermodynamic surface characteristics of multicomponent mouthrinse surfactant solutions at the liquid/vapor interface allowed for the relative *cmc*, surface effectiveness (*γ_cmc_*), area per molecule (*A_molec_*), and surface activity (*σ*) to be determined. The formed adsorption layers turned out to be of the liquid-expanded type. In particular, *σ* is proportional to the partitioning coefficient (*K_P_*) of surfactant molecules between the bulk and surface phases. Lower *K_P_* values correspond to better-soluble compounds in the aqueous phase, thus making them more available for contact with microorganisms in an antibacterial treatment. Surface thermodynamic potentials *H_S_* and *S_S_* revealed a particular mechanism of interfacial film formation and their molecular organization complexity, respectively. Surface wettability energetics parameters, derived from CA studies, for PMMA/mouthrinse systems revealed the following trend in reference to the PMMA/pure water case: *θ*, *θ_A_* and *θ_R_* all ↓, *CAH*↑, *Π*↓, *W_A_*↓, *γ_SV_*↓, $\gamma_{SV}^{p}$↓ and $\gamma_{LV}cos\theta_{A}$↑. The substratum material studied here, characterized by a surface energy of 22.37–30.53 mJ m^−2^, is assumed to induce lower bacterial adhesion as compared to materials with higher surface energies. The ratio: (*γ_LV_* cos *θ_A_*/*W_A_*) ranging from 0.29 (Listerine) to 0.45 (Dentalux + alcohol) is a surface-energetics derived parameter that seems to be a signature of the adhesive strength removal ability of the mouthrinse liquids. The surfactant excesses ratio: $\Gamma_{SL}/\Gamma_{LV}$ was negative and ranged from −1.42 to −0.20, i.e., the range that is characteristic for hydrophobic surface interactions at the *S*/*L* interface. Furthermore, high absolute values of the excess ratio pointed to the formation of multilayered, vertically segregated structures of surfactants composing the mouthrinse mixtures of differentiated surface activity. The pure water mouthrinse dissolution leads to the following wettability parameter variability: *θ_A_* and *θ_R_* ↑, *CAH*↓, *Π*↑, *W_A_*↑, *γ_SV_*~const,$\;\gamma_{SV}^{p}$↑, and $\gamma_{LV}cos\theta_{A}$↓. Finally, the physical properties, CA data, and thermodynamic parameter (*S_S_*) allowed the penetration coefficient (PC = (6.5–9.8)·10^−2^ m s^−1^), and the Marangoni temperature gradient-driven liquid flow speed (*U_S_* = (0.20–0.32)·10^−2^ m s^−1^) speed to be estimated; these factors appear to be important in oral cavity dental plague elimination strategies. This work demonstrates a promising path to control wetting and de-wetting in a polymer-mouth rinse system by properly engineering the liquid–solid interaction at the contact line during advancing and receding movements rather than the entire surface modification.

## Figures and Tables

**Table 1:** Physical, surface, and penetration-spreading characteristics of probe liquids at 22 °C. Experimental uncertainties and standard deviations are given in round brackets (first row).

Liquid	*Γ_LV_* [mN m^−1^] (0.2)	*ρ* [10^3^ kg m^−3^] (0.03)	*μ* [mPa s] (0.05)	pH [-] (0.1)	Product Source
Xerostom	38.7	0.92	1.03	7.2	Bio Cosmetics, Madrid, Spain
Ortho Salvia Dental	39.9	0.97	1.12	7.3	Atos, Warsaw, Poland
Listerine Cool Mint	37.6	0.98	1.32	4.3	Johnson & Johnson Consumer Health, France
Dentalux (alcohol free)	29.7	0.96	1.27	6.0	Cosmolux, Pulheim, Germany
Dentalux (+alcohol)	31.7	0.93	1.10	4.9	Cosmolux, Pulheim, Germany
Eurodont	33.7	0.92	1.05	5.6	Maxim Markenprodukte, Pulheim, Germany
Coca Cola	64.1	0.91	1.01	3.8	Lidl Market
Apple Juice	54.1	1.04	1.12	3.6	Lidl Market
Orange Juice	61.1	1.19	1.21	4.5	Lidl Market
Pure water	72.5	0.89	0.86	6.8	
Saliva	53.6	1.12	1.02	6.8	20-year old healthy volunteer

**Table 2:** Surface adsorptive parameters of mouthrinses at air/liquid interface (*T* = 22 °C). Mean values and standard deviations (given in brackets).

Liquid	*cmc* [*c*/*c*_0_]	*γ_cmc_* [mN m^−1^]	*Γ_max_* [×10^6^ mol cm^−2^]	*A_molec_* nm^2^ molec.^−1^	*|*σ*|* [mN m^−1^]
Xerostom	0.06 (0.01)	40.9 (0.1)	0.42 (0.09)	392.21 (10.15)	210 (16)
Ortho Salvia Dental	0.03 (0.01)	42.7 (0.2)	0.24 (0.08)	839.53 (15.23)	380 (18)
Listerine Cool Mint	0.12 (0.02)	40.0 (0.2)	0.94 (0.09)	170.24 (4.45)	230 (19)
Dentalux (alcohol free)	0.03 (0.01)	31.2 (0.2)	0.50 (0.08)	331.87 (6.23)	810 (32)
Dentalux (+alcohol)	0.03 (0.01)	30.9 (0.2)	0.61 (0.07)	310.61 (6.10)	850 (24)
Eurodont	0.03 (0.01)	32.4 (0.2)	0.91 (0.06)	178.46 (3.78)	1120 (67)
Saliva	0.31 (0.08)	53.7 (0.2)	1.35 (0.08)	12.634 (1.87)	40 (5)

**Table 3:** Surface tension and entropic contributions to the surface enthalpy (following Equations (5) and (6), for probe liquids at *T* = 22 °C (295 K). Experimental uncertainties and standard deviations are given in brackets.

Liquid	*Γ_LV_* [mN m^−1^]	*TS_s_* [mN m^−1^]	*H_s_* [mN m^−1^]
Xerostom	38.7 (0.2)	28.1 (0.2)	66.8 (0.4)
Ortho Salvia Dental	39.9 (0.2)	38.4 (0.2)	78.3 (0.4)
Listerine Cool Mint	37.6 (0.2)	41.3 (0.2)	78.9 (0.4)
Dentalux (alcohol free)	29.7 (0.2)	67.9 (0.2)	97.6 (0.4)
Dentalux (+alcohol)	31.7 (0.2)	62.0 (0.2)	93.7 (0.4)
Eurodont	33.7 (0.2)	37.4 (0.2)	71.1 (0.4)
Saliva	53.6 (0.2)	31.9 (0.2)	85.6 (0.4)
Pure Water	72.5 (0.2)	46.3 (0.2)	118.8 (0.4)
Coca Cola	64.1 (0.2)	52.7 (0.2)	116.8 (0.4)
Orange Juice	61.1 (0.2)	38.0 (0.2)	99.1 (0.4)

**Table 4:** Wettability energetics parameters, for probe liquid–PMMA systems at *T* = 22 °C (295 K) derived from dynamic CA data. Experimental uncertainties and standard deviations are given in brackets.

Liquid	*Θ* [°]	*Θ_A_* [°]	*Θ_R_* [°]	*CAH* [°]	*Π* [mN m^−1^]
Xerostom	41.7 (1)	42.2 (1)	12.1 (1)	30.7 (2)	9.2 (0.4)
Ortho Salvia Dental	43.1 (1)	55.6 (1)	16.7 (1)	38.9 (2)	15.7 (0.6)
Listerine Cool Mint	46.1 (1)	65.2 (1)	18.7 (1)	46.5 (2)	19.8 (0.7)
Dentalux (alcohol free)	39.2 (1)	54.9 (1)	13.1 (1)	41.8 (2)	12.7 (0.5)
Dentalux (+alcohol)	31.9 (1)	43.9 (1)	10.0 (1)	23.9 (2)	4.9 (0.3)
Eurodont	30.2 (1)	55.7 (1)	22.3 (1)	33.4 (2)	12.3 (0.5)
Coca Cola	36.5 (1)	49.2 (1)	13.9 (1)	35.3 (2)	20.3 (0.8)
Apple Juice	54.2 (1)	58.8 (1)	16.9 (1)	41.9 (2)	23.3 (0.9)
Orange Juice	68.8 (1)	74.0 (1)	25.0 (1)	49.0 (2)	27.4 (1.1)
Water	64.7 (1)	76.0 (1)	52.0 (1)	24.0 (2)	26.9 (1.0)
Saliva	72.7 (1)	88.6 (1)	55.3 (1)	33.3 (2)	29.2 (1.2)
Liquid	*W_A_* [mJ m^−2^]	*Γ_SV_* [mJ m^−2^]	*Γ_SV_^d^* [mJ m^−2^]	*Γ_SV_^p^* [mJ m^−2^]	*Γ_LV_* cos*Θ_A_* [mN m^−1^]
Xerostom	67.3 (1.2)	31.5 (1.5)	29.3 (1.3)	2.2 (0.2)	28.7 (0.2)
Ortho Salvia Dental	62.5 (1.1)	27.8 (1.5)	24.5 (1.2)	3.3 (0.3)	22.6 (0.2)
Listerine Cool Mint	53.3 (1.1)	22.5 (1.4)	18.9 (1.1)	3.6 (0.3)	15.6 (0.2)
Dentalux (alcohol free)	50.4 (1.1)	22.4 (1.3)	19.9 (1.1)	2.5 (0.2)	18.4 (0.2)
Dentalux (+alcohol)	58.0 (1.2)	27.8 (1.5)	26.5 (1.4)	1.3 (0.1)	26.3 (0.2)
Eurodont	52.7 (1.1)	23.6 (1.4)	20.6 (1.1)	3.0 (0.3)	18.9 (0.2)
Coca Cola	106.1 (1.6)	48.4 (2.0)	43.8 (1.5)	4.5 (0.5)	41.9 (0.2)
Apple Juice	81.0 (1.4)	35.4 (1.8)	30.7 (1.3)	4.6 (0.5)	27.6 (0.2)
Orange Juice	55.4 (1.1)	22.2 (1.5)	17.7 (1.0)	4.5 (0.5)	11.9 (0.2)
Water	89.5 (1.4)	38.9 (2.0)	27.8 (1.3)	11.1 (0.7)	10.2 (0.2)
Saliva	54.9 (1.1)	21.7 (1.5)	14.1 (1.0)	7.6 (0.5)	1.3 (0.2)

**Table 5:** Wettability parameters for Eurodont-PMMA system (*T* = 22 °C) versus mouthrinse relative concentration $c/c_{0}$; $c_{0}$—initial product concentration, cmc **= 0.032.** Experimental uncertainties and standard deviations are given in brackets.

*c*/*c*_0_ (2%)	*Θ_A_* [°] (1°)	*Θ_R_* [°] (1°)	*Π* [mN m^−1^]	*W_A_* [mJ m^−2^]	*Γ_SV_* [mJ m^−2^]	*Γ_SV_^d^* [mJ m^−2^]	*Γ_SV_^p^* [mJ m^−2^]	*Γ_LV_* cos*Θ_A_* [mN m^−1^]
1	55.8	22.2	12.3 (0.5)	52.7 (1.1)	23.6 (1.4)	20.6 (1.1)	3.0 (0.3)	18.9 (0.2)
0.25	47.9	19.3	8.5 (0.3)	51.8 (1.1)	23.8 (1.4)	21.6 (1.1)	2.3 (0.3)	21.0 (0.2)
0.125	55.9	24.4	11.1 (0.5)	49.6 (1.0)	22.3 (1.4)	19.4 (1.0)	2.9 (0.4)	17.8 (0.2)
0.063	62.9	30.4	13.1 (0.5)	46.9 (1.0)	20.6 (1.3)	17.1 (0.8)	3.5 (0.5)	14.6 (0.2)
**0.032**	**64.8**	**32.3**	**13.6** (0.5)	**46.2** (0.9)	**20.1** (1.3)	**16.5** (0.8)	**3.7** (0.5)	**13.8** (0.2)
0.016	70.2	34.0	16.2 (0.6)	44.4 (0.8)	18.8 (1.2)	14.9 (0.6)	3.9 (0.6)	11.3 (0.1)
0.0078	73.0	50.9	12.0 (0.5)	45.7 (0.9)	20.2 (1.3)	14.8 (0.6)	5.4 (0.7)	10.3 (0.1)
0.0039	82.4	37.3	27.7 (0.8)	47.4 (0.9)	18.3 (1.2)	13.4 (0.5)	4.9 (0.7)	5.5 (0.1)
0.00195	83.9	56.3	22.6 (0.7)	55.8 (1.3)	23.2 (1.4)	15.4 (0.7)	7.8 (0.7)	5.4 (0.1)
0.00098	84.0	63.5	19.8 (0.6)	64.2 (1.5)	27.8 (1.5)	17.7 (0.8)	10.1 (0.7)	6.1 (0.1)
0.00048	88.2	64.3	25.2 (0.8)	64.7 (1.5)	27.1 (1.5)	16.7 (0.8)	10.4 (0.7)	2.0 (0.1)
0.00024	96.5	69.0	32.0 (0.9)	60.3 (1.4)	23.8 (1.4)	13.4 (0.5)	10.5 (0.9)	−7.7 (0.1)

**Table 6:** The $\Gamma_{SL}/\Gamma_{LV}$ ratio (see Equation (13)) for the surfactant water solution/PMMA interfacial system. Experimental uncertainties and standard deviations are given in brackets.

Liquid	*Γ* *_SL_* */Γ_LV_*	Remarks, Data Source
**Mouthrinses**		
Xerostom	−1.23 (0.14)	*c* < *cmc*
Ortho Salvia Dental	−0.20 (0.08)	*c* < *cmc*
Listerine Cool Mint	−1.37 (0.19)	*c* < *cmc*
Dentalux (alcohol free)	−1.21 (0.17)	*c* < *cmc*
Dentalux (+alcohol)	−1.14 (0.24)	*c* < *cmc*
Eurodont	−1.49 (0.23) *c* < *cmc*	−1.81 (0.31) *c* > *cmc*
**Monocomponent model surfactant water solution**		
AOT (dioctyl sodium sulfosuccinate), anionic, double alkyl chains	−0.85 (0.12)	*c* < *cmc* [32]
Tween 20 (polyethylene glycol sorbitan monooleate)	−0.76 (0.15)	*c* < *cmc* [39]
SDS (sodium dodecyl sulphate), anionic	−0.31 (0.09)	*c* < *cmc* [39]
CTAB (cetyltrimethylammonium bromide), cationic	−0. 34 (0.12)	*c* < *cmc* [40,41]
Triton TX-100, nonionic	−0.17 (0.06)	*c* < *cmc* [36,41]
Zwitterionic and cationic gemini surfactants (extended surfactants)	−0.31 (0.01)	(0.34) (0.03)
	*c* < *cmc*	*c* > *cmc*
	[20]	[7]

**Table 7:** Penetration coefficients and Marangoni spreading speeds for selected probe liquid-PMMA systems. Experimental uncertainties are given in brackets.

Liquid	Xerostom	Ortho Salvia Dental	Listerine Cool Mint	Dentalux (Alcohol Free)	Dentalux (+Alcohol)	Eurodont
PC [10^−2^ m s^−1^]	9.8 (0.5)	8.9 (0.5)	6.5 (0.3)	7.4 (0.4)	7.8 (0.4)	7.4 (0.4)
U_S_ [10^−2^ m s^−1^]	0.21 (0.1)	0.23 (0.1)	0.20 (0.1)	0.46 (0.2)	0.32 (0.1)	0.28 (0.1)
Liquid	Coca Cola	Apple Juice	Orange Juice	Pure water	Saliva	-
PC [10^−2^ m s^−1^]	7.2 (0.4)	5.8 (0.3)	6.2 (0.3)	9.8 (0.5)	4.3 (0.2)	-
U_S_ [10^−2^ m s^−1^]	0.41 (0.2)	0.64 (0.3)	0.43 (0.2)	0.80 (0.4)	0.39 (0.2)	-

## Data Availability

Data could be shared on demand.
